# A model of adaptive capacity through collective action in regional disaster mitigation governance in Mamuju Regency, West Sulawesi, Indonesia

**DOI:** 10.4102/jamba.v18i1.2197

**Published:** 2026-10-19

**Authors:** Fadlan Nur Saeni, Muhammad Yunus, Gita Susanti, Badu Achmad, Mohamad Thahir Haning, Muhammad Tang Abdulah

**Affiliations:** 1 Department of Public Administrative Science, Faculty of Social and Political Sciences, Hasanuddin University, South Sulawesi, Makassar, Indonesia

**Keywords:** adaptive capacity, collective action, disaster governance, social learning, disaster risk reduction, process-based model, multihazard risk, Mamuju

## Abstract

Disaster mitigation governance increasingly requires attention to adaptive capacity as a process developed through interaction among actors rather than as a fixed set of resources or institutional attributes. In multihazard settings such as Mamuju Regency, West Sulawesi, this perspective helps explain how actors reorganise in response to disruption. This study examined how collective action contributed to adaptive capacity in local disaster mitigation governance in Mamuju Regency. This interpretive qualitative case study included 35 participants from disaster management agencies, local government institutions, volunteer organisations, and civil society. Data were generated through in-depth interviews, multistakeholder focus group discussions (FGDs), and institutional documents and analysed using thematic coding, interpretive reconstruction, and process tracing. Four interconnected mechanisms were identified: trust formation, adaptive coordination, social learning, and resource mobilisation. Their effects were visible in the reorganisation of communication and coordination during disruption. Adaptive capacity developed unevenly: absorptive capacity was most clearly observed, anticipatory capacity remained emerging, and transformative capacity was developing and conditional on the institutionalisation of learning and collaborative practices. Collective action did not automatically generate adaptive capacity; its contribution depended on whether interaction produced observable and sustained changes in preparedness, learning, coordination, and institutional adjustment.

**Contribution:**

The study provides a process-based explanation linking structural risk conditions, crisis-triggered interaction, collective-action mechanisms, adaptive outcomes, differentiated adaptive capacity, and governance adjustment.

## Introduction

Disaster risk is increasingly understood not simply as a direct consequence of natural hazards, but as the outcome of interactions between biophysical conditions and socially embedded vulnerabilities (Cutter 2021; Lavell et al. 2023; Shi et al. 2020). Patterns of development, resource distribution, institutional capacity, and social relations influence how hazards are experienced and how their consequences unfold. From this perspective, disasters are not isolated external events but manifestations of vulnerabilities produced and reproduced within social and institutional systems over time (Cinner et al. 2018).

Mamuju Regency in West Sulawesi illustrates this complexity. The region is characterised by recurrent, overlapping geological and hydrometeorological hazards. The 2021 earthquake showed how physical disruption can quickly spill over into social, economic, and institutional systems, especially when communication, mobility, public services, and coordination are affected simultaneously (Anastasia, Alimuddin & Arifin 2021; Laode et al. 2025; Widiastuti et al. 2025). These conditions make Mamuju a relevant setting for examining how local actors respond and reorganise under persistent multihazard risk.

Despite the increasingly complex character of disaster risk, mitigation governance in many settings remains dominated by sectoral and technocratic approaches. Risks are often classified by individual hazards, leading to interventions centred on specific technical measures rather than on the relationships among hazards, vulnerability, institutions, and social processes (Drakes & Tate 2022; Hochrainer-Stigler et al. 2023; Hulke & Revilla Diez 2020; Scolobig, Komendantova & Mignan 2017). Such approaches help identify and manage specific hazards, but they offer a more limited explanation of how actors reorganise relationships, information, and resources when established arrangements are disrupted.

A related limitation concerns how adaptive capacity has been conceptualised. Many studies assess adaptive capacity through indicators such as education, infrastructure, access to information, economic resources, and institutional capability (Chapagain et al. 2025; Johnson et al. 2020). These approaches provide important comparative measures, but they can also portray capacity primarily as something that individuals, communities, or institutions already possess. Less attention has been given to how capacity develops through interaction, learning, coordination, and adjustment in response to changing conditions.

The literature on collective action offers a complementary perspective. Classical approaches emphasise the challenges of cooperation in the pursuit of shared interests, while institutional and contemporary studies highlight the importance of trust, communication, norms, networks, and repeated interaction in sustaining collaboration (Amigo & Echavez 2023; Hulke & Revilla Diez 2020; Olson 1971; Ostrom 1990; Rayamajhee & Bohara 2021; Wannewitz, Petzold & Garschagen 2023). Collective action has also been linked to social learning and the capacity of actors to respond jointly to environmental and disaster-related challenges (Johnson et al. 2020; Soubry, Sherren & Thornton 2020). However, it is still commonly treated as a facilitating condition for adaptation rather than as a process through which adaptive capacity itself may be generated.

This separation leaves an important analytical question unresolved: How does collective interaction actually produce adaptive capacity in practice? Recent studies have increasingly recognised that disaster preparedness and adaptation depend on relationships across institutions, networks, and levels of governance rather than on isolated organisational resources (Cvetković et al. 2021; McNaught et al. 2024; Nikkanen, Räsänen & Juhola 2024; Wannewitz et al. 2023). Other work has similarly shown that adaptive responses are shaped by social relations, institutional arrangements, behavioural adjustment, and the ability of actors to reorganise under changing risk conditions (Berkhout 2024; Imperiale & Vanclay 2021; Ro & Garfin 2023). These developments create a basis for examining adaptive capacity as an evolving social process rather than solely as a measurable endowment.

Against this background, this study examines how adaptive capacity develops through collective action in local disaster mitigation governance. It advances a process-based model that links structural risk conditions, crisis-triggered interaction, collective-action mechanisms, observable adaptive outcomes, and differentiated dimensions of adaptive capacity. The empirical analysis highlights four mechanisms: Trust formation, adaptive coordination, social learning, and resource mobilisation.

The study contributes to the literature in two respects. Theoretically, it brings adaptive-capacity and collective-action perspectives into a common analytical framework by treating collective action as a capacity-producing process rather than merely as a supportive condition (Canadas et al. 2023; Fila, Fünfgeld & Lorenz 2024; Hulke & Revilla Diez 2020). Empirical evidence from Mamuju shows how these mechanisms operate under multihazard risk and how their effects differ across absorptive, anticipatory, and transformative capacity. This distinction provides a process-based explanation for why effective collective response during emergencies does not necessarily translate immediately into sustained preparedness or institutional transformation.

## Research methods and design

### Study design and setting

This study employed an interpretive qualitative case study design to examine how adaptive capacity develops through collective action in disaster mitigation governance. The study was conducted in Mamuju Regency, West Sulawesi, Indonesia, which was purposively selected because of its multihazard setting and experience of the 2021 earthquake. The case provided an appropriate context for examining coordination, collective response, learning, and institutional adjustment following disaster disruption.

### Participants and data collection

Participants were selected purposively based on their involvement in disaster mitigation, preparedness, emergency response, communication, or community-based disaster activities. The study included 35 unique participants: three Badan Penanggulangan Bencana Daerah (BPBD) officials, 25 representatives of local government institutions outside BPBD, three representatives of volunteer organisations (including Radio Antar Penduduk Indonesia [RAPI]), and four civil society representatives (including Karampuang Foundation). Table 1 presents the participant composition.

**TABLE 1:** Participant categories and basis for inclusion.

Participant category	Number of participants	Basis for inclusion
Badan Penanggulangan Bencana Daerah officials	3	Direct involvement in disaster management policy, coordination, and mitigation programmes
Local government representatives	25	Institutional role in local disaster governance and public service coordination
Volunteer organisations, including Radio Antar Penduduk Indonesia	3	Direct role in emergency communication, evacuation, and field coordination
Civil society organisations, including Karampuang Foundation	4	Involvement in community support, advocacy, local capacity building, and disaster response evaluation

**Total**	**35**	**-**

Data were collected through in-depth interviews, multistakeholder focus group discussions (FGDs), and institutional documents. Interviews and FGDs explored experiences of coordination, communication disruption, trust, learning, resource mobilisation, preparedness, and institutional adjustment. Documentary materials were used to contextualise and cross-check participant accounts. Secondary data from the Indonesia Disaster Risk Index (IRBI) and BNPB were used descriptively to establish the structural risk context of West Sulawesi and Mamuju and were not treated as a separate quantitative component.

### Data analysis

The data were analysed using a process-oriented approach that combined thematic coding, interpretive reconstruction, and process tracing. The analysis examined interview, FGD, and documentary materials for recurring patterns related to crisis disruption, coordination, trust formation, social learning, resource mobilisation, and governance adjustment. Comparisons across actor groups identified common and contrasting experiences.

The analysis then traced how crisis-related disruption intensified interaction among actors and how collective-action mechanisms were linked to observable changes in communication, organisation, learning, and response. These patterns were interpreted in relation to **absorptive, anticipatory, and transformative capacity**. The process-based model emerged from the relationships identified in the empirical data rather than being imposed as a predetermined framework. Triangulation across interviews, FGDs, and documents, along with attention to contradictory and constraining evidence, strengthened the credibility of the interpretation.

### Ethical considerations

Ethical clearance to conduct this study was granted by the Faculty of Social and Political Sciences, Hasanuddin University (No. 09782/UN4.1.14/PT.01.04/2025). All participants provided informed consent prior to data collection. Participant confidentiality was maintained throughout the research process. Personal identifiers were excluded from interview and focus group transcripts and from the presentation of findings, while potentially identifying information was omitted from quoted material to protect participant anonymity.

## Results

### Structural risk conditions

West Sulawesi remained in the high disaster-risk category in 2024, with an IRBI score of 155.69. Although the index declined from 190.88 in 2015, the trend was not linear, showing periods of decline, stability, and slight increase (see Figure 1). The province faces multiple hazards, including earthquakes, tsunamis, floods, landslides, forest and land fires, drought, extreme waves, and coastal abrasion. Flooding was reported as the most frequently occurring disaster in West Sulawesi in 2024.

**FIGURE 1:**
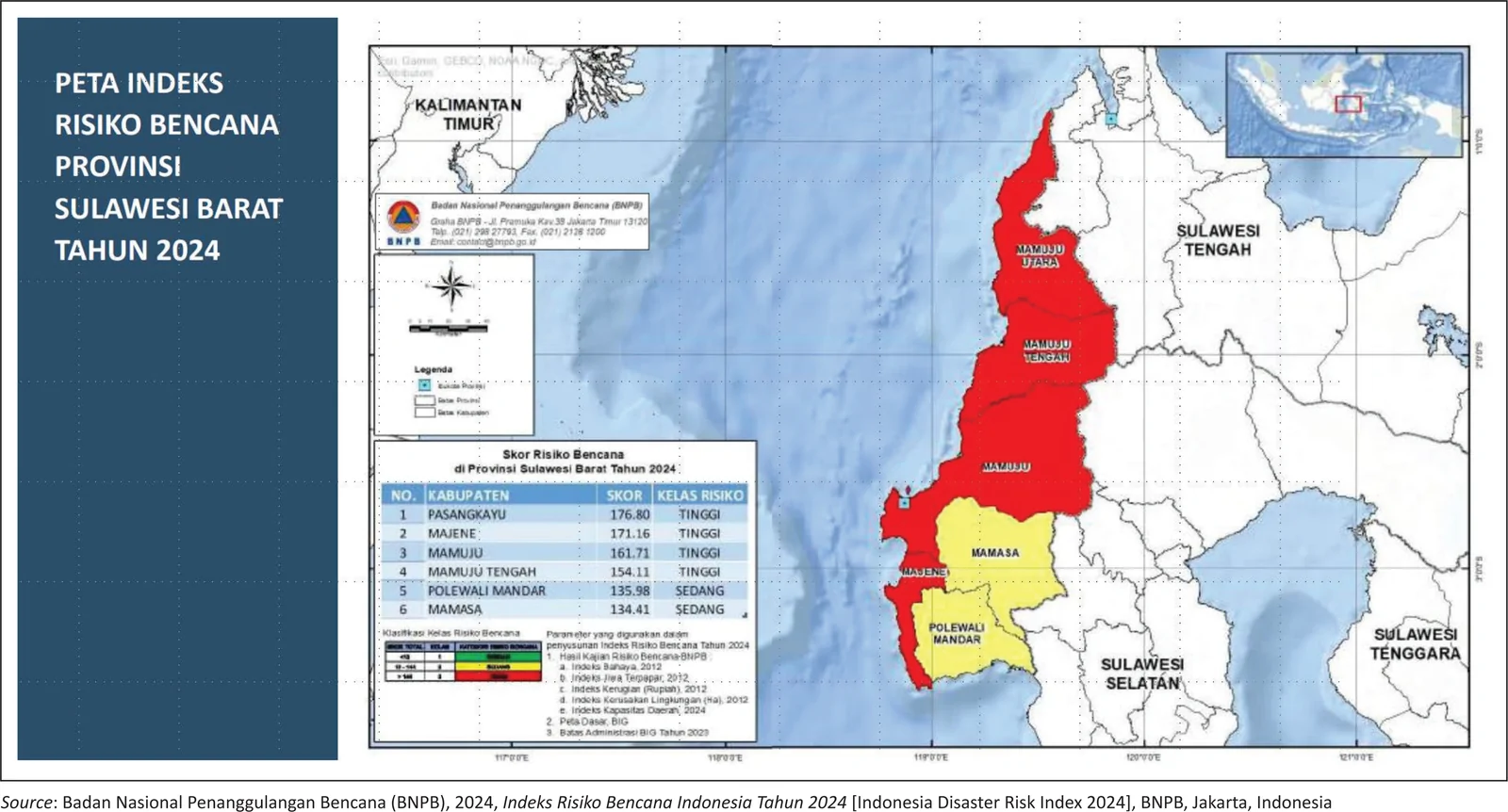
Disaster risk index map of West Sulawesi province for 2024.

Risk levels varied across the six regencies. In 2024, Pasangkayu (176.80), Majene (171.16), Mamuju (161.71), and Central Mamuju (154.11) remained in the high-risk category, while Polewali Mandar (135.98) and Mamasa (134.41) were classified as medium risk.

Mamuju showed a fluctuating trajectory, declining from 200.40 in 2015 to 136.13 in 2017 before increasing to 161.71 in 2022 and remaining at that level through 2024. This pattern confirms that Mamuju continues to experience a persistent high-risk condition despite changes in its index over time.

The temporal pattern shows that disaster risk across West Sulawesi did not decline uniformly from 2015 to 2024 (see Figure 2). While several regencies recorded lower risk-index values over time, Mamuju followed a more fluctuating trajectory and remained within the high-risk category in 2024. The detailed variation across regencies is presented in Table 2.

**FIGURE 2:**
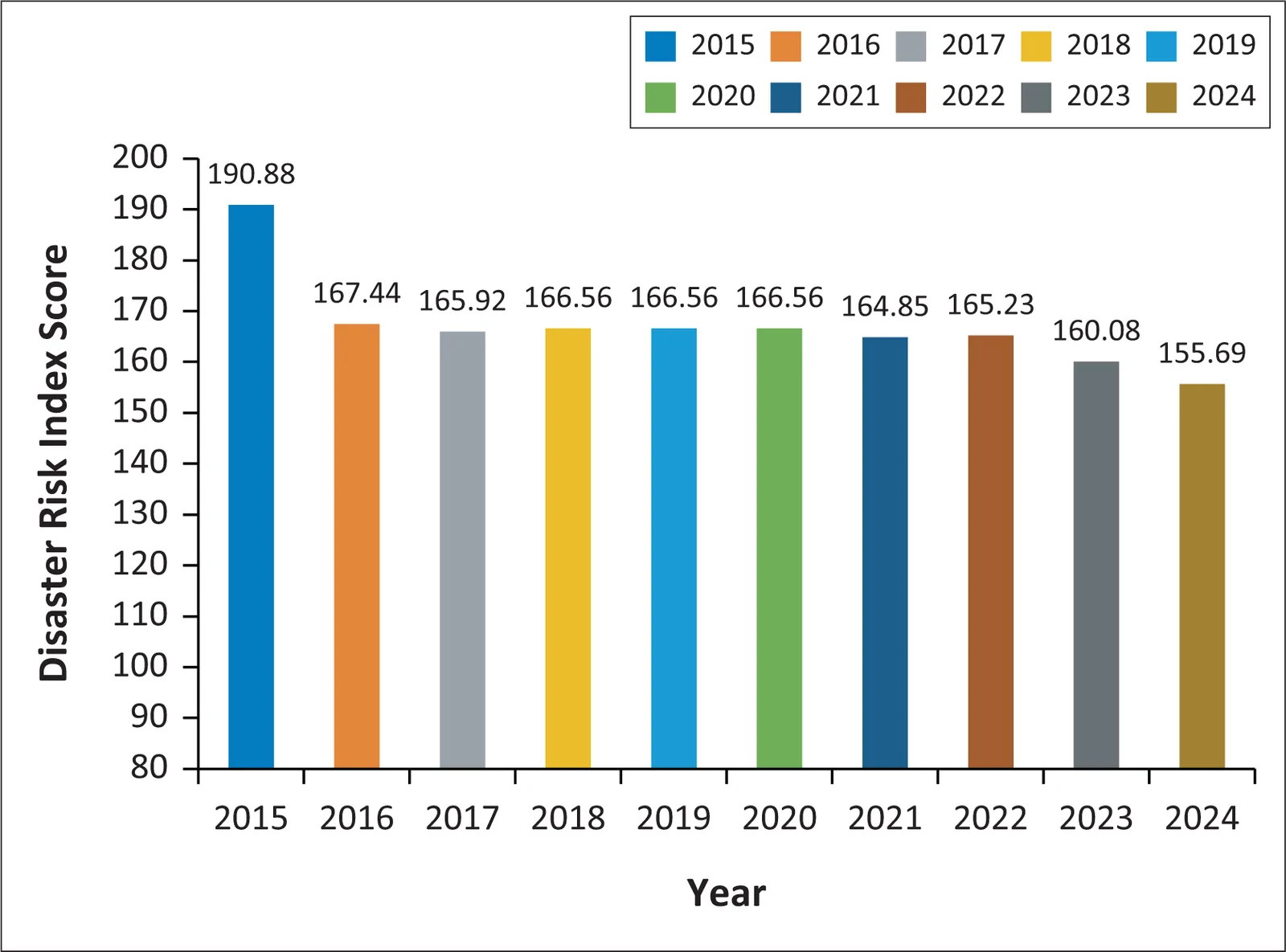
Temporal dynamics of disaster risk index in West Sulawesi province (2015–2024).

**TABLE 2:** Disaster risk index values for West Sulawesi province, 2015–2024.

No	Regency or city	2015	2016	2017	2018	2019	2020	2021	2022	2023	2024	Risk class 2024
1	Pasangkayu	176.8	176.8	176.8	176.8	176.8	176.8	176.8	176.8	176.8	176.8	High
2	Majene	221.2	221.2	221.2	216.08	216.08	216.08	217.62	208.3	186.83	171.16	High
3	Mamuju	200.4	137.03	136.13	145.13	145.13	145.13	145.13	161.71	161.71	161.71	High
4	Central Mamuju	-	-	-	-	-	-	161.71	161.71	159.33	154.11	High
5	Polewali Mandar	202	148.2	141.48	140.81	140.81	140.81	140.81	135.81	135.93	135.98	Medium
6	Mamasa	154	154	154	154	154	154	147.02	147.02	139.87	134.41	Medium

*Source:* Badan Nasional Penanggulangan Bencana (BNPB), 2024, *Indeks Risiko Bencana Indonesia Tahun 2024* [Indonesia Disaster Risk Index 2024], BNPB, Jakarta, Indonesia

Risk levels also varied across the six regencies. In 2024, Pasangkayu (176.80), Majene (171.16), Mamuju (161.71), and Central Mamuju (154.11) were classified as high risk, whereas Polewali Mandar (135.98) and Mamasa (134.41) were classified as medium risk. This variation indicates that the recorded risk profile is not uniform across West Sulawesi.

Mamuju showed a particularly fluctuating trajectory. Its risk index declined from 200.40 in 2015 to 137.03 in 2016 and 136.13 in 2017, increased to 145.13 between 2018 and 2021, and rose again to 161.71 in 2022, where it remained through 2024. By comparison, Majene showed a longer-term decline from 221.20 to 171.16, while Pasangkayu remained unchanged at 176.80 throughout the period. Polewali Mandar and Mamasa recorded lower values by 2024 and were classified as medium risk.

### Crisis-triggered interaction

The earthquake exposed weaknesses in coordination and communication arrangements in Mamuju. Participants described the initial response as fragmented, particularly when substantial assistance arrived before clear coordination and distribution mechanisms were established. One civil society participant recalled ‘a significant level of confusion during the first days after the earthquake’, with assistance arriving in large quantities but not being effectively organised.

Focus group discussion participants similarly noted that disaster response could become sporadic, with institutions sometimes operating independently. However, Pentahelix collaboration reportedly functioned more effectively during emergency response than during the predisaster phase. The crisis therefore increased the need for government institutions, volunteer organisations, civil society groups, and communities to coordinate their roles under disrupted conditions.

### Collective action mechanisms and observable adaptive outcomes

Four mechanisms emerged consistently from the interviews and FGDs: Trust formation, adaptive coordination, social learning, and resource mobilisation.

Adaptive coordination was most evident in emergency communication. When internet and cellular networks were disrupted, high frequency (HF) and very high frequency (VHF) radio communication provided an alternative channel. Radio Antar Penduduk Indonesia supported this communication function, while BPBD retained its formal coordination role. Together, they enabled communication to continue despite disruptions to conventional infrastructure.

Trust developed through repeated interaction and the perceived reliability of participating organisations. The visible presence of organised volunteer actors helped communities recognise that assistance remained available, while participants stressed that institutions could not undertake disaster management ‘moving on their own’. Nevertheless, cooperation remained partly dependent on individual commitment and had not been fully supported by stable institutional arrangements.

Social learning was reflected in accumulated disaster experience, routine training, risk information, and local knowledge. Participants nevertheless reported a gap between knowledge and preparedness. Information from agencies such as the Indonesian Agency for Meteorology, Climatology, and Geophysics (BMKG) was available, but community understanding and response remained uneven, indicating that technical information still required more effective translation into local practice.

Resource mobilisation also depended on coordination. Assistance was available following the earthquake, but effective distribution required shared information, clear responsibilities, and agreement on priority needs. Participants particularly emphasised the importance of preventing assistance from becoming concentrated in only certain locations.

Together, these mechanisms contributed to observable changes in communication, coordination, learning, and the organisation of available resources.

### Differentiated adaptive capacity

The findings indicate that adaptive capacity developed unevenly across its three dimensions. Absorptive capacity was the most clearly observed, as actors reorganised communication and coordination during disruption, particularly through the complementary roles of BPBD, RAPI, and other emergency actors. Anticipatory capacity remained emerging, supported by disaster experience, training, risk information, and local knowledge, but constrained by weaknesses in public risk literacy, predisaster coordination, and translating lessons into routine preparedness.

Transformative capacity was developing and conditional. Participants recognised the need for stronger communication arrangements, updated planning documents, cross-sectoral coordination, regulatory support, and broader participation. However, limited funding, outdated risk assessments, dependence on individual leadership, and gaps in including vulnerable groups indicate that institutional change remains incomplete. Overall, the capacity to absorb immediate disruption was more evident than the capacity to anticipate future risks or achieve longer-term institutional transformation.

### Governance and preparedness adjustment

Previous disaster experience had begun to influence preparedness priorities. Participants reported continued training for government personnel and volunteers, greater recognition of alternative communication systems, and efforts to strengthen coordination across institutions. Discussions also mentioned HF and/or VHF communication arrangements, disaster communication support guidance, and the development of a Disaster Communication Support Centre (Pusdikom).

These adjustments remain incomplete. Participants identified limited funding, an outdated Disaster Risk Assessment (*Kajian Risiko Bencana*, KRB), weak regulatory arrangements, uneven predisaster coordination, and insufficient attention to women, older people, and persons with disabilities in preparedness planning.

Governance adjustment in Mamuju is therefore still ongoing. The findings show that disaster experience has strengthened awareness and coordination practices, but their continuity depends on incorporation into routine planning, budgeting, communication arrangements, and institutional procedures.

Taken together, the results show that persistent structural risk and crisis-related disruption intensified interaction among actors, strengthened particular forms of collective response, and produced uneven development of adaptive capacity. These empirical relationships provide the basis for the analytical synthesis developed in the following section.

### Model development: Adaptive capacity through collective action

The findings suggest that adaptive capacity in disaster governance develops through interaction rather than through the presence of resources or institutional arrangements alone. Building on the relationships identified across the empirical findings, this study proposes a Process-Based Model of Adaptive Capacity through Collective Action. The model connects structural risk conditions, crisis-triggered interaction, collective-action mechanisms, observable adaptive outcomes, differentiated adaptive capacity, and subsequent governance and preparedness adjustment, as presented in Figure 3.

**FIGURE 3:**
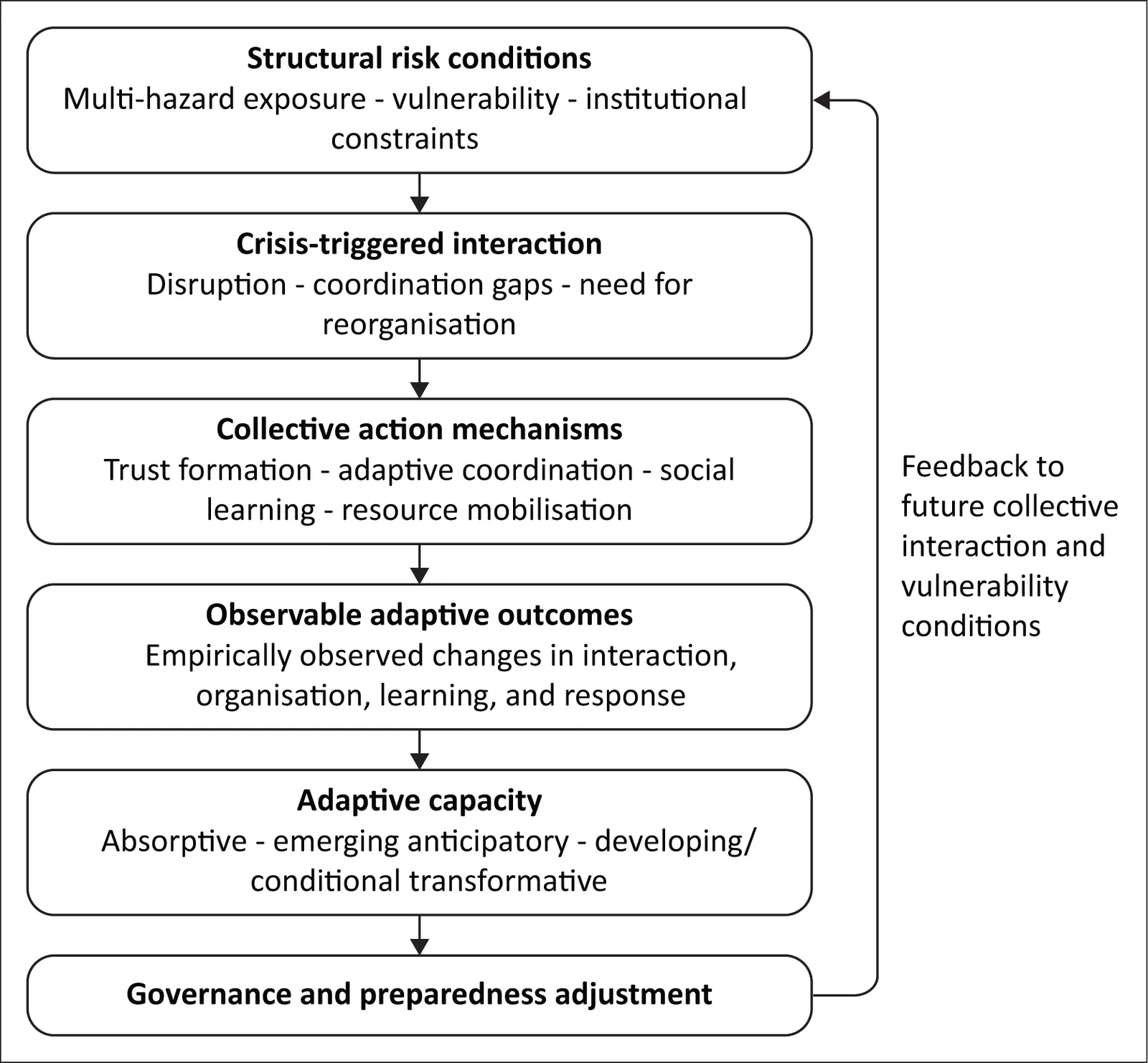
Process-based model of adaptive capacity through collective action in disaster mitigation governance.

Structural risk conditions provide the setting in which collective responses become necessary, but they do not by themselves produce adaptive capacity. When a crisis disrupts established communication, coordination, and organisational routines, actors must reconsider how they interact and organise their response. In this setting, crisis-triggered interaction creates the conditions through which trust formation, adaptive coordination, social learning, and resource mobilisation become operational. These mechanisms are interconnected and may reinforce one another during collective response.

Their contribution to adaptive capacity becomes visible when interaction produces changes in practice. These include continuity or reorganisation of essential functions, clearer coordination among actors, learning from previous experience, and more organised use of available resources. Such outcomes provide the empirical link between collective action and adaptive capacity. The findings further show that this capacity does not develop uniformly. Absorptive capacity was the most clearly observed, while anticipatory capacity remained emerging and transformative capacity was developing and conditional on whether learning and collective practices became embedded in more durable institutional arrangements.

The process does not end with the immediate response. Experience generated through collective action may influence subsequent preparedness priorities, coordination arrangements, institutional procedures, and other governance adjustments. These adjustments can, in turn, shape how actors interact and how institutional and social vulnerability is addressed when future hazards occur. The feedback represented in the model therefore concerns changes in preparedness, interaction, and vulnerability conditions rather than changes in the underlying physical hazards.

Taken together, the model conceptualises adaptive capacity as an evolving process that begins with structural risk conditions and is activated through crisis-triggered interaction. These interactions give rise to collective-action mechanisms, which in turn generate observable adaptive outcomes and contribute to differentiated forms of adaptive capacity. Over time, these capacities may influence governance and preparedness adjustments, while the resulting institutional and social changes can shape future patterns of collective interaction and vulnerability.

This process-based model emphasises that collective action does not produce adaptive capacity uniformly or automatically. Its contribution depends on whether interaction leads to observable changes in practice and whether those changes are sustained beyond the immediate crisis through continued learning, coordination, and institutional adjustment.

## Discussion

The findings show that adaptive capacity in Mamuju develops unevenly through collective interaction rather than existing as a fixed set of institutional or material resources. This is most apparent in the contrast between emergency response and predisaster preparedness. Multi-actor collaboration became more functional when actors faced an immediate shared problem, whereas coordination outside the emergency period remained less consistent. This helps explain why absorptive capacity was the most clearly observed, while anticipatory capacity remained emerging and transformative capacity was developing and conditional. The distinction extends approaches that assess adaptive capacity primarily through socioeconomic, institutional, informational, or material attributes by showing that these resources gain adaptive value only when actors can connect and reorganise them in practice (Chapagain et al. 2025; Johnson et al. 2020; Vallury et al. 2022).

One clear example is the emergency communication arrangement involving BPBD and volunteer communication networks such as RAPI. When internet and cellular systems were disrupted, HF and VHF radio provided an alternative way to maintain communication, while BPBD continued to exercise its formal coordination role. The significance of this example lies not in the availability of radio technology itself, but in the complementary relationship between formal authority and distributed operational capability. Communication could continue despite disruption because different actors could perform distinct but connected functions. This supports recent work on adaptive and collaborative disaster governance, which emphasises flexibility, distributed capability, and inter-organisational networks where uncertainty cannot be managed through hierarchical arrangements alone (McNaught et al. 2024; Nikkanen et al. 2024). In Mamuju, however, adaptive coordination did not require replacing formal authority; it emerged through a combination of formal leadership and flexible operational support.

This experience also clarifies the role of trust in collective action. Trust was not simply a resource that actors possessed before cooperation occurred. It developed as organisations demonstrated reliability and became recognisable partners during disruption. This reciprocal relationship is consistent with Ostrom’s (1990) emphasis on communication, repeated interaction, and trust in sustaining collective action, as well as evidence from post-earthquake settings showing the importance of social capital and trust where formal institutional capacity is under pressure (Rayamajhee & Bohara 2021). The Mamuju findings add that trust can be both an outcome and an enabling condition of collective action: Cooperation provides opportunities for actors to demonstrate reliability, while that demonstrated reliability makes subsequent cooperation easier. At the same time, dependence on personal commitment and the absence of stronger binding arrangements indicate that relational trust has developed more readily than institutionalised trust.

The distinction between experience and preparedness is equally important. Government actors, volunteers, and communities have accumulated knowledge through previous disasters, routine training, formal risk information, and local experience. Yet they have not consistently converted this knowledge into anticipatory practice. Uneven public engagement with risk information, weaknesses in predisaster coordination, and difficulty maintaining updated strategic documents illustrate the gap between learning from past events and systematically preparing for future ones. This supports studies that treat social learning as a collective process in which experience becomes adaptive only when it is exchanged, interpreted, and translated into changed practice (Hulke & Revilla Diez 2020; Soubry et al. 2020; Wannewitz et al. 2023). The Mamuju case therefore suggests that experience alone does not constitute anticipatory capacity. Its adaptive value depends on whether lessons are preserved through planning, risk communication, exercises, institutional memory, and routine coordination.

Resource mobilisation provides a similar lesson. The arrival of substantial assistance after the earthquake did not automatically produce an effective response; participants reported difficulties in coordinating distribution and preventing resources from becoming concentrated in particular locations. This illustrates why adaptive capacity cannot be inferred simply from the quantity of resources available. Resources become useful when actors can share information about needs, coordinate responsibilities, and organise their distribution. This interpretation aligns with studies showing that adaptive capacity depends on the social and institutional relations through which available resources are accessed and used (Fila et al. 2024; Imperiale & Vanclay 2024). Collective action therefore changes the analytical question from what resources are available to how available resources are organised under changing conditions.

These findings also explain why transformative capacity remains conditional. Mamuju shows evidence of adjustment: Training continues, alternative communication arrangements are receiving greater attention, cross-sector coordination is increasingly recognised, and previous disaster experience is influencing preparedness priorities. However, an outdated Disaster Risk Assessment (KRB), limited strategic funding, regulatory gaps, uneven predisaster coordination, dependence on individual leadership, and shortcomings in including vulnerable groups indicate that these lessons have not yet produced comprehensive institutional transformation. Similar studies of institutional adaptive capacity and disaster preparedness emphasise that learning becomes durable only when it is incorporated into governance arrangements rather than remaining dependent on temporary projects or individual actors (Nikkanen et al. 2024; Ro & Garfin 2023). Transformative capacity in Mamuju should therefore be understood as developing rather than achieved.

The broader theoretical contribution of the study lies in showing that collective action is not merely a favourable condition surrounding adaptive capacity. It is part of the process through which capacity is produced. Trust formation makes cooperation more feasible; adaptive coordination connects formal and informal capabilities under disruption; social learning allows experience to inform subsequent action; and resource mobilisation converts available assets into organised response. Their effects, however, are neither automatic nor uniform. Collective action may strengthen the ability to absorb an immediate shock without producing a corresponding transformation in preparedness institutions. Likewise, learning may improve awareness without being translated into formal planning. This uneven development is important because it avoids treating absorptive, anticipatory, and transformative capacities as stages that necessarily follow one another.

For disaster governance, the practical implication is that effective emergency collaboration must extend beyond the crisis period. The Mamuju experience suggests that preparedness would benefit from maintaining routine interaction between government agencies, volunteer communication networks, civil society organisations, and communities; preserving communication redundancy; updating risk and contingency documents; strengthening mechanisms for translating technical risk information into locally meaningful communication; and incorporating vulnerable groups more systematically into preparedness processes. The objective is not to formalise every spontaneous practice, but to retain those relationships and lessons that proved useful during disruption while reducing dependence on individual initiative. In this sense, stronger adaptive capacity requires not only resources and formal institutions, but governance arrangements capable of sustaining interaction, learning, and coordination between disaster events.

## Conclusion

This study examined how adaptive capacity develops through collective action in disaster mitigation governance in Mamuju Regency, a region characterised by persistent multihazard risk. The findings show that adaptive capacity is not simply determined by the availability of institutional, social, or material resources. It develops through interactions among actors when those resources are connected through trust, coordination, learning, and organised mobilisation in response to changing conditions.

The evidence reveals an uneven pattern of adaptive capacity. Absorptive capacity was the most clearly observed, particularly in the ability of government and volunteer actors to reorganise communication and maintain essential functions during disruption. Anticipatory capacity remained emerging, as experience, training, and risk information had not yet been consistently translated into routine preparedness. Transformative capacity was developing and conditional, constrained by gaps in regulation, strategic planning, funding, institutional continuity, and inclusive participation. Effective collaboration during a crisis therefore does not automatically result in stronger preparedness or lasting institutional change.

The study contributes a process-based understanding of the relationship between collective action and adaptive capacity. Rather than treating collective action as an external condition that merely supports resilience, the findings show how interaction among actors can itself become a mechanism through which adaptive capacity is produced. At the same time, this process remains contingent: Lessons and collaborative practices generated during emergencies need to be retained and incorporated into routine governance if they are to influence future preparedness and vulnerability conditions.

For disaster governance in Mamuju and comparable multihazard settings, the findings point to the importance of sustaining collaboration beyond the emergency phase. Strengthening routine cross-sector coordination, maintaining redundant communication arrangements, translating disaster experience into updated planning and preparedness practices, and ensuring wider participation can help convert crisis-based cooperation into more durable adaptive capacity. The central challenge is therefore not only to mobilise actors when disaster occurs, but to preserve and institutionalise the relationships and learning that make collective action effective between disaster events.
